# The Sleep, Recovery, and Nutrition Characteristics of Elite Adolescent Athletes

**DOI:** 10.3390/sports13020050

**Published:** 2025-02-10

**Authors:** Lorcán Mason, James Connolly, Lydia E. Devenney, Karl Lacey, Jim O’Donovan, Maria Faulkner, Rónán Doherty

**Affiliations:** 1 Department of Tourism and Sport, Atlantic Technological University Donegal, Port Road, F92 FC93 Letterkenny, Ireland; 2 School of Computing, Engineering and Intelligent Systems, Ulster University, Northland Rd., Londonderry BT48 7JL, UK; 3 School of Psychology and Counselling, The Open University, Walton Hall, Milton Keynes MK7 6AA, UK; 4 School of Health and Human Performance, Dublin City University, DCU Glasnevin Campus, Collins Avenue Extension, Dublin 9, D09 Y8VX Dublin, Ireland; 5 Sport Ireland Institute, National Sport Campus, Abbotstown, Dublin 15, D15 Y52H Dublin, Ireland

**Keywords:** sleep, recovery, dietary behaviours, elite sports, youth

## Abstract

Background: Elite sport participation creates a significant burden on adolescent athletes due to demanding training schedules, high training intensities, and the complexity of puberty. As such, an athletes’ ability to effectively balance stress and recovery is essential for their athletic performance and requires appropriate management throughout the competitive season. This research aimed to investigate: (i) the quantity, quality, and timing of sleep; (ii) general and sport-specific nutrition knowledge; (iii) recovery practices; and (iv) the relationships between sleep, nutrition, and recovery practices in elite adolescent athletes. Methods: A total of 51 athletes completed a battery of previously validated and reliable questionnaires which investigated their sleep characteristics, nutrition knowledge, and recovery practices. Results: Statistically significant moderate correlations were observed between their Pittsburgh Sleep Quality Index (PSQI) global score, Sleep Difficulty Classification (SDC), and Recovery Stress Questionnaire for Athletes (REST-Q Sport) scales, with small significant correlations observed between the SDC and REST-Q recovery scales. Participants had lower scores in both their sport (36.10 ± 12.13) and total (40.25 ± 11.18) nutrition knowledge compared to their general nutrition knowledge (49.53 ± 16.46). Moderate levels of general (1.50 ± 0.86) and sport-specific (1.57 ± 0.85) stress and high levels of general (3.92 ± 0.74) and sport-specific (3.72 ± 0.96) recovery were demonstrated. Furthermore, 94% reported their sleep quality to be “fairly good” (*n* = 30) or “very good” (*n* = 18). Conclusions: These findings highlight the necessity of further investigating the effect of educational strategies on improving sleep, nutrition, and recovery knowledge in athletes to aid recovery and dietary behaviours.

## 1. Introduction

An athlete’s ability to effectively balance stress and recovery is essential to their performance and requires appropriate management throughout the competitive season [1,2,3,4]. The implementation of fundamental recovery strategies related to sleep, dietary behaviours, and recuperation periods are pivotal for athletes’ performance, health, and well-being [2,4,5,6]. Moreover, the implementation of these strategies varies depending on activity type, the training status of the athlete, and the specific outcome measures of the sport [6,7,8]. Different sports require different approaches; team sports including association football and Gaelic games, with more frequent matches, may benefit more from increased recovery than individual sports like swimming, where major competitions are less frequent [6,7,8,9]. Furthermore, recovery methods should be periodized, strategically implemented, and adapted to the athlete’s specific needs and the nature of their stressors [6,7,9]. This involves adjusting recovery strategies based on the phase of training, competition schedule, and individual responses [6,7,9].

Nonetheless, athletes often report poor sleep practices, with a significant percentage experiencing insufficient sleep due to factors such as stress, travel, and the timing of training and competitions [6,7,10]. The importance of sleep cannot be overstated, as it plays a restorative role in metabolic regulation, immune system function, and cognitive and physical performance, with inadequate sleep linked to increased injury risk and impaired recovery [11,12,13,14]. Nutrition is another critical component of recovery, with specific dietary behaviours shown to improve sleep quality and quantity [10,15]. For instance, high-glycaemic-index carbohydrates, tryptophan-rich protein, tart cherry juice, and kiwifruit have been identified as beneficial for enhancing sleep parameters [10,15]. Pre-sleep nutrition, particularly protein consumption, has been suggested to positively affect muscle protein synthesis and overnight recovery, although its impact on the duration and quality of sleep, next-day performance, and recovery warrants further investigation [15,16,17,18]. Recovery practices among athletes vary, with hydration, nutrition, sleep, and rest being the most preferred and effective strategies [6,7,8]. The implementation of recovery strategies is influenced by athletes’ knowledge, beliefs, the sources of information they rely on, and their specific sports and competition levels [6,19,20].

Despite the established roles of sleep and nutrition in the recovery process [10,15,21,22], limited research has been conducted in adolescent athletes [23]. Moreover, elite sport participation creates a significant burden on adolescent athletes due to demanding training schedules, high training intensities, and the complexity of puberty [24,25,26,27]. Moreover, the incidence of injury is prevalent among adolescent athletes, occurring during both training (~1.4–6.4 per 1000 h) and competition (~22.4 per 1000 h) [27,28,29,30]. Additionally, adaptations to sleep regulation occur during adolescence [31], which increase sleep pressure tolerance [32], causing a circadian phase delay [33], thus compounding the bioregulatory, societal, and psychosocial pressures present during adolescence [34,35], creating an inadequate environment for recovery in adolescent athletes [23,36,37]. Furthermore, research demonstrates that nutrition knowledge in adolescent athletes is poor, resulting in inadequate dietary behaviours to support recovery [38,39,40]. Both sleep and nutrition have been emphasised as modifiable factors that can be manipulated to support recovery, mitigate the risk of injury, and improve performance [6,7,10,21,22,41].

Therefore, the aims of this research were to investigate (i) the quantity, quality, and timing of sleep; (ii) general and sport-specific nutrition knowledge; (iii) the recovery practices of elite adolescent athletes; and (iv) the relationships between sleep, nutrition, and recovery practices in elite adolescent athletes.

## 2. Methods

### 2.1. Participants

This study utilised a convenience sampling method for recruitment purposes, whereby participants were recruited if they matched specified criteria not met by the general population [42,43]. A sample (*n* = 51) of elite youth team-sport athletes were recruited (Table 1). In accordance with previous athlete-specific literature, they were defined as elite if they were (a.) receiving support/funding through the international carding scheme and/or (b.) members of a national/professional team or a recruitment/academy squad and/or (c.) nationally ranked in their sport [44]. Participants were excluded if they were (a.) aged >19 years, (b.) were classified as <Tier 3 on the participant classification framework [45], or (c.) had a diagnosed sleep disorder.

### 2.2. Procedure

**Figure 1:**
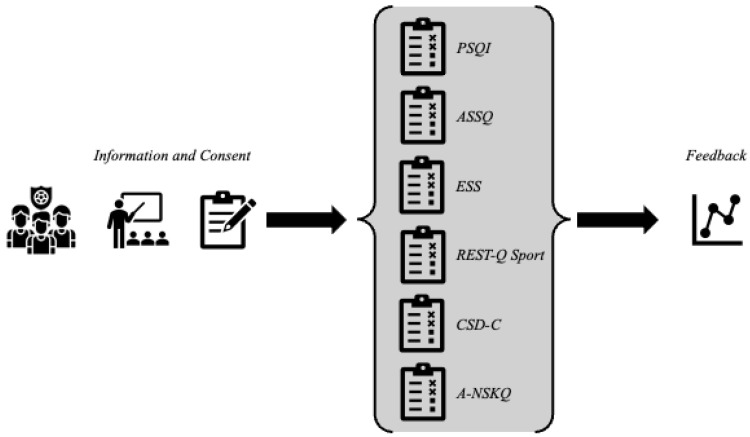
Schematic of study design.

The elite team-sport athletes were invited to partake in online questionnaires. All procedures were approved by the Institute Research Ethics Committee of Atlantic Technological University Donegal (09/11/22) and Garda Vetting was completed by the principal investigator prior to data collection. After reading the participant information sheet, the athletes’ parent(s)/guardian(s) were invited to provide informed consent online via Qualtrics^xm^ (https://www.qualtrics.com/). In addition, the athletes were given an age-appropriate explanation of the research and invited to provide their assent. Once both parental consent and the athlete’s assent were received, the athletes were then asked to complete the online survey, which consisted of a battery of previously validated, reliable, and widely used questionnaires assessing their sleep, recovery, and nutritional characteristics. The online survey was available between 27/03/23 and 31/04/23, with participants completing each questionnaire in the same order within a thirty-minute timeframe to ensure consistency during this period. Following completion of the questionnaires, participants received a debrief sheet with details of how they could contact the researcher if they wished to receive feedback from the survey (Figure 1).

### 2.3. Measures

In the initial section of the questionnaire, the athletes filled out their demographic data. Athletes recorded their age (yrs), gender, height (m), body mass (kg), sport(s) played, phase of season (pre-season, competition, or off-season), normal training time (before 8 a.m., 8 a.m. to 5 p.m., or after 5 p.m.) and the duration of training/competitions per week (mins).

### 2.4. Pittsburgh Sleep Quality Index (PSQI)

A valid and reliable (Cronbach’s alpha = 0.83; test–retest reliability r = 0.85) self-reported questionnaire, the Pittsburgh Sleep Quality Index (PSQI) was used to assess the participants’ sleep quality and disturbances over the prior month [46]. The PSQI comprises 19 items grouped into seven equally weighted scoring components (0–3), whereby component scores consist of subscales for subjective sleep quality, latency, total duration, regulation, interruption, dysfunction during the day, and medication [46,47]. The PSQI is a reliable measure of sleep quality, with high diagnostic sensitivity and specificity, 89.6% and 86.5%, respectively, in discerning ‘good’ and poor’ sleepers [46,47,48,49]. Global scoring is measured via summating the component scores in the range of 0–21, with greater values indicating poorer sleep [47,48,50]. Global scores > 8 have been used to indicate “poor sleep” within the literature; however, more conservative scores of ≥5 have been used in athletes to indicate poor sleep, potentially due to the increased sleep needs of this population [11,14,51].

### 2.5. Athlete Sleep Screening Questionnaire (ASSQ)

The clinically valid Athlete Sleep Screening Questionnaire (ASSQ) is used to identify candidates for further sleep assessments [52,53] and is a reliable (r = 0.86) sleep assessment tool displaying acceptable levels of intra-individual consistency [52,53]. The 16-item questionnaire is categorised into six key areas of sleep assessment: total duration, insomnia characteristics, quality, chronotype, sleep breathing disorders, and travel disturbances [52,53]. Sleep problems are categorised based on a scaled Sleep Difficulty Classification (SDC) score (0–17), with higher scores inferring greater severity [52,53].

### 2.6. Epworth Sleepiness Scale (ESS) for Children and Adolescents

The Epworth Sleepiness Scale (ESS) for Children and Adolescents is a reliable and validated (Cronbach’s alpha = 0.73, test–retest reliability r = 0.89) eight-item questionnaire that measures subjective daytime sleepiness [54,55,56]. Participants are questioned on how likely they are to fall asleep while partaking in the following activities: (a.) sitting and reading, (b.) watching television, (c.) sitting inactive in a public place, (d.) being a passenger in a car for at least 1 h, (e.) lying down to rest or nap in the afternoon, (f.) sitting and talking to someone, (g.) sitting quietly alone after lunch, and (h.) sitting and eating a meal [54,55,56]. Each question is scored on a four-point Likert scale of 0 to 3 [54,55,56]. Total scores range from 0 (unlikely to fall asleep in any situation) to 24 (a high chance of falling asleep in all situations). Excessive daytime sleepiness is defined as an ESS global score of ≥10 [54,56].

### 2.7. Recovery Stress Questionnaire for Athletes (REST-Q Sport)

The REST-Q Sport is a validated 52-item subjective questionnaire sensitive to the stress and recovery incurred by sports and general lifestyles [57,58]. The REST-Q Sport consists of nineteen scaled sections comprising seven general stress components (general stress, emotional stress, social stress, conflicts/pressure, fatigue, lack of energy, and physical complaints), five general recovery components (success, social recovery, physical recovery, general well-being, and sleep quality), three sport-specific stress components (disturbed breaks, burnout/emotional exhaustion, and fitness/injury), and four sport-specific recovery components (fitness/being in shape, burnout/personal accomplishments, self-efficacy, and self-regulation) [57]. These nineteen scaled sections assess the athletes’ previous three days and nights using a six point Likert scale (0 = Never to 6 = Always) [57], with the mean scores of the sub-scale components being combined to give a total score for each of the four predominant scales of stress and recovery (general stress, general recovery, sport-specific stress, and sport-specific recovery) [57,58]. High scores in the stress components indicate the presence of greater stress, while high scores in the recovery components indicate the presence of greater recovery [57,58].

### 2.8. Consensus Sleep Diary—Core (CSD-C)

The Consensus Sleep Diary—Core is a standardised sleep diary used for both research and clinical purposes [11,59]. Eight components are included in the sleep diary, including reported bed time, sleep initiation time, sleep onset duration, the number and durations of awakenings, final waking time and rising time, and a Likert scale for the self-reported rating of sleep quality [60], which is completed for three nights (1 training day (TD), 1 competition day (CD), and 1 rest day (RD)) [11]. There is also a comments section where participants can record specific comments about each night’s sleep. The data collected is then used to calculate indicators of sleep continuity, including the total time in bed (TIB), total sleep time (TST), sleep onset latency (SOL; time from light out to N1), wakefulness after sleep onset (WASO; amount of time awake after sleep onset), number of awakenings (NoA), and sleep efficiency (SE; the ratio of TST:TIB) [60].

### 2.9. The Abridged Nutrition for Sport Knowledge Questionnaire (A-NSKQ)

The abridged Nutrition For Sport Knowledge Questionnaire (A-NSKQ) was used as a validated tool to assess general and sports nutrition knowledge [61,62,63]. The 35-item A-NSKQ contains two sub-sections [62,63] with several key areas of focus, including body composition and weight management, general nutrition, supplementation, sport nutrition, and alcohol [62,63]. Answers are summated, giving a ‘total score’, ‘general nutrition knowledge score’, and a ‘sport nutrition knowledge score’ [62,63]. The A-NSKQ is reliable (r = 0.7) and has construct validity, having been validated against the Rasch model [62,63].

### 2.10. Data Analysis

Descriptive and inferential statistics were utilised for our analysis [43], with Excel used for data cleaning and Jamovi version 2.3 (The Jamovi Project, Sydney, Australia) and R version 4.4.1 [43,64], utilising the ‘gtsummary’ [65], ‘ggstatsplot’ [66], and ‘stats’ packages [64], used for analysis. Descriptive statistics and frequency distributions were used to present the findings [43]. The normality and distribution of the data were assessed using histograms, Q-Q plots, and Shapiro–Wilk tests [43]. Differences between data were analysed by performing a one-way ANOVA (normal data) and Kruskal–Wallis one-way ANOVA (non-normal data) [43]. Descriptive analysis observed the general characteristics of the sample in terms of central tendencies and variability [43,67]. Inferential analysis facilitated an understanding of relationships, differences, and cause and effect between the variables collected [43,67]. Data are reported as a mean ± standard deviation (SD), unless otherwise stated. The alpha level was set to *p* ≤ 0.05 to determine significance and the effect size (ES) for pairwise comparisons was calculated using eta squared (η^2^) and classified as follows: trivial (η^2^: <0.20), small (0.21–0.60), moderate (0.61–1.20), large (1.21–2.00), or very large (>2.00) [68].

## 3. Results

### 3.1. The Abridged Nutrition for Sport Knowledge Questionnaire (A-NSKQ)

**Figure 2:**
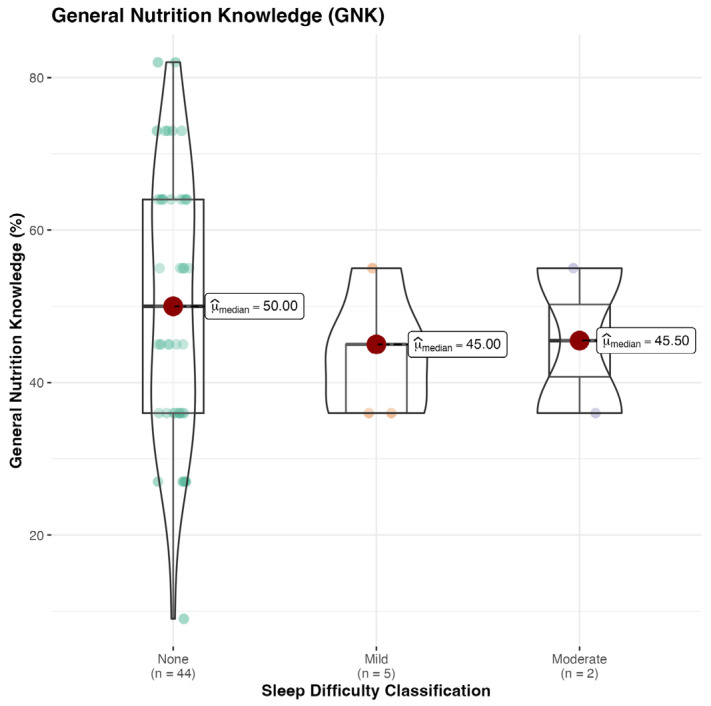
General nutrition knowledge (GNK) score, %.

**Figure 3:**
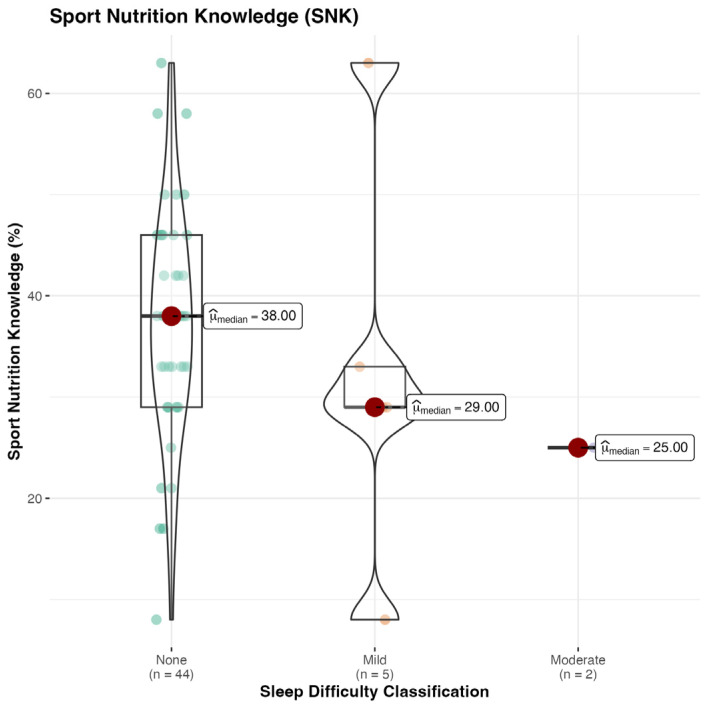
Sport nutrition knowledge (SNK) score, %.

**Figure 4:**
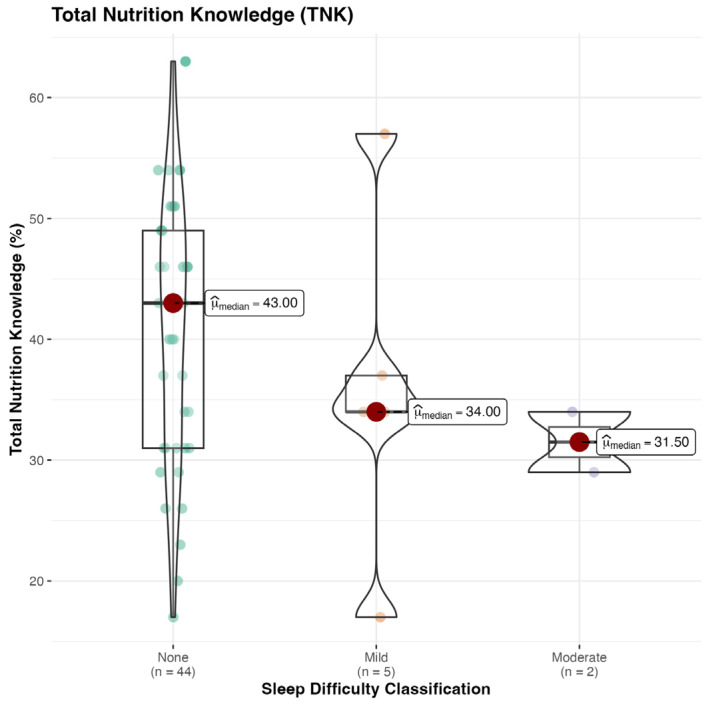
Total nutrition knowledge (TNK) score, %.

The general descriptive statistics of the Abridged Nutrition for Sport Knowledge Questionnaire (A-NSKQ) are presented in Table 2. A Kruskal–Wallis one-way ANOVA demonstrated significant differences between the groups for the Sleep Difficulty Classification (SDC). No significant differences were found between groups for general, sport or total nutrition knowledge (Figure 2, Figure 3 and Figure 4).

### 3.2. Pittsburgh Sleep Quality Index (PSQI)

The Pittsburgh Sleep Quality Index (PSQI) global score results are presented in Table 2. Significant differences were determined by way of a Kruskal–Wallis one-way ANOVA between groups (SDC). Significant differences were observed for the measures contained in the sleep quality, sleep latency, total sleep time (hrs), sleep efficiency (%SE), and global score components (Table 3). For measures of sleep quality, significant differences were observed between SDCs (χ^2^(2) = 10.40, *p* = 0.006), with a mean sleep quality rating of 2.00 ± 1.41 for moderate SDC, 1.40 ± 0.55 for mild SDC, and 0.59 ± 0.50 for no SDC. The effect size, eta squared (η^2^), was 0.21, which is classified as small (η^2^ < 0.20). For measures of sleep latency, significant differences were observed between SDCs (χ^2^(2) = 13.28, *p* = 0.001), with a mean sleep latency of 2.50 ± 0.71 for moderate SDC, 2.00 ± 0.00 for mild SDC, and 0.95 ± 0.71 for no SDC, with a small effect (η^2^ = 0.27). Similarly, significant differences were observed between SDC groups for total sleep time (TST) (χ^2^(2) = 20.38, *p* ≤ 0.001, η^2^ = 0.41) and sleep efficiency (%SE) (χ^2^(2) = 6.58, *p* = 0.037, η^2^ = 0.13). Furthermore, significant differences (χ^2^(2) = 14.10, *p* ≤ 0.001, η^2^ = 0.28) were observed for measures of the PSQI global score between SDCs (moderate: 10.50 ± 2.12; mild: 7.20 ± 2.17; none: 3.66 ± 1.83) with a small effect.

### 3.3. Recovery Stress Questionnaire for Athletes (REST-Q Sport)

A Kruskal–Wallis one-way ANOVA established no significant difference in the general recovery scores between groups. Furthermore, when taking the general recovery items into account, a small significant difference was found between the none (4.60 ± 1.05) and moderate (2.00 ± 1.41) SDC groups in terms of sleep quality (χ^2^(2) = 9.74, *p* = 0.008, η^2^ = 0.19). No significant differences were found for the general stress, sport-specific stress, and sport-specific recovery scales between SDC groups (Table 4). General stress scale scores were similar in each of the none (1.40 ± 0.80), mild (1.96 ± 1.18), and moderate (2.64 ± 0.40) SDC groups. Moreover, similar scores were found between the groups for sport-specific stress (none: 1.51 ± 0.78; mild: 1.52 ± 1.03; moderate: 2.96 ± 1.24) and sport-specific recovery (none: 3.80 ± 0.86; mild: 3.25 ± 1.69; moderate: 3.13 ± 0.88) scales.

### 3.4. Consensus Sleep Diary—Core

The subjective sleep characteristics of the Consensus Sleep Diary—Core for training (TD), competition (CD) and rest day (RD) days are presented in Table 5. A one-way ANOVA demonstrated significant differences between day types for total sleep time (TST) and time in bed (TiB). Significant differences were observed between training and rest days (TD: 9.47 ± 1.31 vs. RD: 10.52 ± 1.30) for TST (F(2, 99.83) = 8.31; *p* ≤ 0.001). Furthermore, significant differences were found between both training and competition days (TD: 9.69 ± 1.20 vs. CD: 10.17 ± 1.22) and training days and rest days (TD: 9.69 ± 1.20 vs. RD: 10.89 ± 1.31) for TIB (F(2, 99.87) = 11.47; *p* ≤ 0.001). No significant differences were found between day types for sleep onset latency (SOL), number of awakenings, wake after sleep onset (WASO), and sleep quality (Table 5).

Pearson’s correlation (r) was used to assess the strength of the relationships between the ASSQ Sleep Difficulty Classification, A-NSKQ, PSQI, and REST-Q variables. Statistically significant moderate correlations were observed between the PSQI global score, SDC, and REST-Q scales, with Pearson’s r values ranging from 0.3 to 0.67 (Table 6). Furthermore, small significant correlations were observed between the Sleep Difficulty Classification (SDC) and REST-Q recovery scales (Table 6). Moreover, no significant correlations were observed between A-NSKQ scores, PSQI global scores, SDCs, or REST-Q scales (Table 6).

## 4. Discussion

This research aimed to investigate the recovery practices and the quantity, quality, and timing of sleep in elite adolescent athletes via the REST-Q, PSQI, ASSQ, ESS and Consensus Sleep Diary—Core; to investigate the general and sport-specific nutrition knowledge of these athletes via the A-NSKQ; and, finally, to explore the relationships between these variables. The results demonstrated that no significant correlations were observed between the A-NSKQ score, PSQI global score, SDC, and REST-Q scales (Table 6). However, statistically significant moderate correlations were observed between the PSQI global score, SDC, and REST-Q scales, while significant but small correlations were observed between the SDC and REST-Q recovery scales (Table 6). Given sleep is regarded as a necessary restorative process for optimising recovery [6,69], it is imperative to address the challenges to sleep that persist during adolescence [34,35]. Moreover, for training adaptations to occur, fundamental recovery strategies must be in place, including obtaining sufficient sleep, ensuring adequate nutritional intake, and implementing adequate recovery periods within the training cycle [2,4,5,6].

### 4.1. Nutrition

In the present study, poor overall knowledge of nutrition was observed, with the sample scoring < 50% in all knowledge domains. Participants had lower scores in both sport (36.10 ± 12.13) and total (40.25 ± 11.18) nutrition knowledge compared to general nutrition knowledge (49.53 ± 16.46). Moreover, when athletes were grouped into SDCs, no significant differences were observed between individuals in terms of their sport, total and general nutrition knowledge scores (%). As established within the literature, nutrition knowledge is paramount to informing nutritional behaviours that support healthy physiological function [39,70]. Research investigating nutrition knowledge is sparse in adolescent athletes and a consensus is yet to be established [39,71,72,73,74]. In athletes, high levels of nutrition knowledge are required to establish healthy dietary behaviours and inform eating habits that support recovery [39,72,74,75,76]. If individuals possess inadequate levels of nutrition knowledge, their nutritional intake may be negatively impacted by poor dietary choices and decreased dietary quality [38]. This in turn may negatively impact the recovery processes in athletes and, importantly, their adolescent growth and development [39,74,75,76,77]. Furthermore, research indicates that adolescent athletes possess poor levels of nutrition knowledge, leading to inadequate dietary intakes [38,39,40]. Moreover, when compared to their adult counterparts, lower levels of nutrition are present in adolescents, suggesting that nutritional deficiencies and inadequate fuelling are present in this population [38,39,40]. Thus, based on these findings and research highlighting the importance of providing accurate and tailored nutrition education [37,70,78], providing education that focuses on evidence-based recommendations whilst emphasising the importance of nutrition for fuelling performance and the specific dietary behaviours shown to improve sleep quality and quantity is warranted [10,15,70].

### 4.2. Sleep

The mean PSQI global score was 4.27 ± 2.47, with 63% of participants (*n* = 32) identified as good sleepers and 37% (*n* = 19) presenting with poor sleep quality. Furthermore, 94% of the sample reported their sleep quality to be “fairly good” (*n* = 30) or “very good” (*n* = 18). This is corroborated by the CSD-C, as only 15% of participants rated their sleep quality as “poor” (12%; *n* = 19) or “very poor” (3%; *n* = 4). This contrasts with previous research in athletes, which has reported greater mean PSQI global scores at or above the cut-off (≥5) [7,11,51,79]. Moreover, recent studies have reported poor sleep quality in adolescent athletes [48,80,81,82], corroborating results found within athletic populations. Research indicates that adolescent athletes experience poor sleep quality attributed to the bioregulatory, societal, and psychological pressures present during adolescence [34,35], in conjunction with sporting factors such as the timing of training, demanding competitive schedules, high training loads, and performance anxiety [24,27,30,83,84]. The contrast between our findings and previous studies may be explained by several factors, including the benefits from favourable environmental or socio-cultural influences, such as structured schedules or supportive living arrangements, which promote better sleep hygiene [23,80,82,85]; a lack of objectivity in fully capturing nuanced sleep disturbances, such as fragmented sleep or circadian misalignment, which are common in athletic populations [7,11,51]; and the influence of perception and bias, which may not be accurately accounted for to generate one’s true response [86,87]. Furthermore, the contrasting results found in this study may be attributed to the athletic population examined. Research has proposed that due to training and competition schedules, team sport athletes report later wake times, greater time in bed, and greater sleep durations [51]. Additionally, 80% of participants reported their PSQI total sleep time (TST) as >7 h per night, with 20% reporting 6–7 h per night. Interestingly, when considering the TST of the CSD-C, significant differences were observed between training and rest days (TD: 9.47 ± 1.31 vs. RD: 10.52 ± 1.30), which are in line with current recommendations [88]. However, research suggests a tendency toward the overestimation of TST in athletic populations [7,51,89]. Furthermore, the optimal TST is specific to the individual and their environment [35,90,91], and research argues for a greater TST in athletic populations compared to current guidelines [92].

### 4.3. Recovery

The recovery–stress balance is essential for the progressive development of the physical qualities of an athlete to improve their performance [93]. This is achieved through balancing the applied training dose and the appropriate time-course for adequate recovery to facilitate adaptations [94,95]. In the current study, participants demonstrated moderate levels of general (1.50 ± 0.86) and sport-specific (1.57 ± 0.85) stress. Moreover, participants also demonstrated high levels of general (3.92 ± 0.74) and sport-specific (3.72 ± 0.96) recovery. As stated, the athletes’ ability to effectively balance stress and recovery is essential to their athletic performance and requires appropriate management throughout their competitive season [1,2,3,4,96]. Previous research has indicated that injury risk is heightened when athletes report higher levels on the injury subscale and a poor sleep quality rating [7,97]; however, this is not the case in the present study, with participants reporting lower ratings on the injury subscale (2.00 ± 1.05) and good sleep quality (4.35 ± 1.25). Furthermore, research has highlighted the impact of psychological stress (r = 0.27, 80% CI 0.20–0.37) and a history of stressors (r = 0.13, 80% CI 0.11–0.15) on injury [5,7], emphasising the importance of an athlete’s ability to appropriately manage their stressors to reduce their risk of degraded performance and injury [1,2,3,4,5,7,96]. As recovery is a vital component of the training cycle [2,95,96], it is imperative that athletes are educated on the importance of addressing modifiable components of their recovery, including their training load, sleep, nutrition, and psychological stress [2,4,5,6,96]. This is particularly pertinent to adolescent athletes due to the psychological, physiological, and environmental stressors present during adolescence [34,35]. Moreover, the importance of this research, in light of the balance between training and stress, cannot be understated as recovery plays a vital role in the dissipation of fatigue in athletes, thus emphasising the importance of modifiable factors. Most pertinently, nutrition and sleep can aid in the application of the appropriate training dose to induce adaptations [2,6,7,9,36]. These findings highlight the need for dynamic training load adjustments based on recovery–stress assessments. For example, athletes exhibiting elevated stress or inadequate recovery may benefit from a reduced training intensity or increased focus on recovery strategies, such as active recovery sessions, nutritional support, and stress management techniques [6,7,9].

### 4.4. Limitations

As with any self-reported measurement, conscious bias and measurement error exist and are inherent risks that must be stated due to the subjective nature of the results obtained [43,86,98,99]. Perception is an individual cognitive process that can be influenced by non-physiological variables that may not accurately reflect one’s true response [86]. However, self-reported measures play an important role in athlete monitoring since they are inexpensive, non-invasive, and easy to administer to a large population [11,100,101]. Evidence also suggests that subjective measurements may be more responsive and reliable at detecting internal responses than some quantifiable measures [100,101,102,103]. This study utilised measurements that are reliable and have been validated in athletic populations to mitigate the limitations of these subjective measurements. However, future research would benefit from the addition of actigraphy or polysomnography measures to provide a more accurate reflection of the data acquired, offering a greater global assessment [11,14,100,101]. Furthermore, it is important to note the limitations of the A-NSKQ in capturing dietary behaviours, as this tool acts as an assessment of nutrition knowledge and does not evaluate actual dietary intake [61,62,63]. Given these limitations, future research should consider using alternative or complementary tools (Food Frequency Questionnaires, Food Diaries, or 24-Hour Dietary Recalls) to assess an athlete’s dietary behaviours [38]. Moreover, sleep diaries have been reported to be a more accurate measure of sleep than sleep questionnaires [11]; however, this study may be limited in terms of the data collection of the sleep diary, as only one training day, one competition day, and one rest day were assessed. This was chosen to limit questionnaire fatigue and cognitive bias [98,104]; however, this failed to meet the 1-week duration recommended when administering sleep diaries [11,59,105,106]. Additionally, the short duration of the data collection period may limit the generalisability of the findings to longer-term behaviours [11,59,105,106]. Extending the monitoring period in future studies could provide a more comprehensive understanding of athletes’ habitual practices and their variability.

## 5. Conclusions

This study demonstrated no significant correlations between A-NSKQ scores, PSQI global scores, SDCs, or REST-Q scales. However, statistically significant moderate correlations were observed between PSQI global scores, SDCs, and REST-Q scales, while small significant correlations were observed between the SDC and REST-Q recovery scales. Additionally, moderate levels of general (1.50 ± 0.86) and sport-specific (1.57 ± 0.85) stress and high levels of general (3.92 ± 0.74) and sport-specific (3.72 ± 0.96) recovery were observed. Interestingly, 63% (*n* = 32) of the sample were identified to be good sleepers, which contrasts with previous findings [7,11,51,79,107,108]. Moreover, poor overall nutrition knowledge was observed, with the present sample scoring <50% in all knowledge domains.

Research has indicated that providing education to athletes is an effective strategy for improving their sleep hygiene [85,109,110,111] and dietary behaviours [40,70,78,112] to support recovery. Collectively, these findings highlight the necessity of further investigation into the effect of educational strategies on improving sleep, nutrition, and recovery knowledge in athletes to aid their recovery [85,109,110,111] and dietary behaviours [40,70,78,112]. Thus, this research provides evidence that steps must be taken to improve athletes’ knowledge of the fundamental aspects of recovery [6,14,70,78,85,109,110,111,112].

## Figures and Tables

**Table 1:** Participant characteristics *n* = 51 (means ± standard deviations (SDs)).

Characteristic	Overall ^1^
Age (years)	15.67 ± 1.61
Height (m)	1.70 ± 0.99
Body Mass (kg)	60.31 ± 8.86
Years of Participation	9.12 ± 2.40
Sport Activity Duration per Week (mins)	419.22 ± 121.46

^1^ Mean ± SD.

**Table 2:** Participant-level descriptive statistics from the Abridged Nutrition for Sport Knowledge Questionnaire (A-NSKQ), Pittsburgh Sleep Quality Index (PSQI), and Recovery Stress Questionnaire for Athletes (REST-Q Sport) (means ± standard deviations (SD)).

Characteristic	Overall ^1^
GNK Score (%)	49.53 ± 16.46
SNK Score (%)	36.10 ± 12.13
TNK Score (%)	40.25 ± 11.18
Global PSQI	4.27 ± 2.47
General Stress Score	1.50 ± 0.86
General Recovery Score	3.92 ± 0.74
Sport-Specific Stress	1.57 ± 0.85
Sport-Specific Recovery	3.72 ± 0.96

^1^ Mean ± SD. GNK = general nutrition knowledge; SNK = sport nutrition knowledge; TNK = total nutrition knowledge; PSQI = Pittsburgh Sleep Quality Index.

**Table 3:** Pittsburgh Sleep Quality Index (PSQI) Kruskal–Wallis results.

	χ^2^	df	*p*	ε^2^
Sleep Quality	10.40	2	0.006	0.21
Sleep Onset Latency (SOL)	13.28	2	0.001	0.27
Total Sleep Time (hrs)	20.38	2	<0.001	0.41
% Sleep Efficiency (%SE)	6.58	2	0.037	0.13
Global Score	14.10	2	<0.001	0.28

**Table 4:** Recovery Stress Questionnaire for Athletes (REST-Q Sport) (means ± standard deviations (SD)).

Characteristic	Overall ^1^	None ^1^ (*n* = 47)	Mild ^1^ (*n* = 5)	Moderate ^1^ (*n* = 4)	*p*-Value ^2^
Stress	0.96 ± 0.97	0.88 ± 0.97	1.30 ± 0.97	2.00 ± 0.00	0.088
Emotional Stress	1.62 ± 0.89	1.58 ± 0.89	1.60 ± 0.89	2.50 ± 0.71	0.3
Social Stress	1.48 ± 1.04	1.50 ± 1.03	1.00 ± 1.27	2.25 ± 0.35	0.2
Conflicts/Pressure	1.69 ± 1.24	1.48 ± 1.09	3.00 ± 1.70	3.00 ± 0.00	0.029
Fatigue	1.87 ± 1.47	1.72 ± 1.40	2.20 ± 1.44	4.50 ± 0.00	0.053
Lack of Energy	1.64 ± 1.20	1.45 ± 0.95	3.00 ± 2.18	2.25 ± 1.77	0.2
Somatic Complaints	1.25 ± 1.07	1.18 ± 1.01	1.60 ± 1.56	2.00 ± 1.41	0.6
Success	3.15 ± 0.99	3.18 ± 0.99	2.60 ± 0.89	3.75 ± 1.06	0.3
Social Relaxation	4.77 ± 0.92	4.81 ± 0.82	4.40 ± 1.78	5.00 ± 0.00	>0.9
Somatic Relaxation	3.03 ± 1.03	3.10 ± 1.04	2.60 ± 0.96	2.50 ± 0.71	0.4
General Well-Being	4.27 ± 1.01	4.36 ± 0.90	3.60 ± 1.82	4.00 ± 0.00	0.5
Sleep Quality	4.35 ± 1.25	4.60 ± 1.05	3.10 ± 1.29	2.00 ± 1.41	0.008
Disturbed Breaks	1.09 ± 0.81	1.07 ± 0.81	0.90 ± 0.80	2.00 ± 0.00	0.2
Burnout/Emotional Exhaustion	1.61 ± 1.19	1.54 ± 1.08	1.45 ± 1.41	3.50 ± 2.12	0.2
Fitness/Injury	2.00 ± 1.05	1.91 ± 0.95	2.20 ± 1.52	3.38 ± 1.59	0.3
Fitness/Being in Shape	3.98 ± 1.09	4.09 ± 0.97	3.25 ± 1.87	3.38 ± 1.24	0.3
Burnout/Personal Accomplishment	3.38 ± 0.98	3.41 ± 0.94	3.45 ± 1.37	2.50 ± 0.71	0.4
Self-Efficacy	3.51 ± 1.00	3.57 ± 0.89	3.10 ± 1.75	3.38 ± 1.59	0.7
Self-Regulation	4.00 ± 1.17	4.13 ± 1.07	3.20 ± 1.95	3.25 ± 0.00	0.2
General Stress	1.50 ± 0.86	1.40 ± 0.80	1.96 ± 1.18	2.64 ± 0.40	0.12
General Recovery	3.92 ± 0.74	4.01 ± 0.65	3.26 ± 1.22	3.45 ± 0.21	0.2
Sport-Specific Stress	1.57 ± 0.85	1.51 ± 0.78	1.52 ± 1.03	2.96 ± 1.24	0.2
Sport-Specific Recovery	3.72 ± 0.96	3.80 ± 0.86	3.25 ± 1.69	3.13 ± 0.88	0.4

^1^ Mean ± SD. ^2^ Kruskal–Wallis rank sum test.

**Table 5:** Consensus Sleep Diary—Core (means ± standard deviations (SD)).

	TD	CD	RD
TST (hrs)	9.47 ± 1.31 ^b^ [95% CI: 9.1, 9.8]	10.01 ± 1.43 [95% CI: 9.6, 10]	10.52 ± 1.30 [95% CI: 10, 11]
TIB (hrs)	9.69 ± 1.20 ^c^ [95% CI: 9.4, 10]	10.17 ± 1.22 [95% CI: 9.8, 11]	10.89 ± 1.31 ^a^ [95% CI: 11, 11]
SOL (min)	15.98 ± 13.27 [95% CI: 12, 20]	18.53 ± 14.71 [95% CI: 14, 23]	18.04 ± 14.67 [95% CI: 14, 22]
WASO (min)	2.78 ± 5.76 [95% CI: 1.2, 4.4]	3.14 ± 6.22 [95% CI: 1.4, 4.9]	2.71 ± 5.55 [95% CI: 1.1, 4.3]
NoA	0.41 ± 0.75 [95% CI: 0.20, 0.62]	0.55 ± 0.94 [95% CI: 0.28, 0.81]	0.43 ± 0.88 [95% CI: 0.18, 0.68]
Sleep Quality Rating	3.12 ± 0.62 [95% CI: 2.9, 3.3]	3.10 ± 0.81 [95% CI: 2.9, 3.3]	3.37 ± 0.80 [95% CI: 3.1, 3.6]

TST (hrs) = total sleep time; TIB (hrs) = time in bed; SOL (min) = sleep onset latency; WASO (min) = wake after sleep onset; NoA = number of awakenings; TD = training day; RD = rest day; CD = competition day. Note: ^a^ denotes significance (*p* < 0.05) between CD and RD; ^b^ denotes significance (*p* < 0.001) between TD and RD; ^c^ denotes significance (*p* < 0.001) between TD and RD.

**Table 6:** Strength of relationships between ASSQ Sleep Difficulty Classification, A-NSKQ, PSQI, and REST-Q variables.

		TNK Score (%)	PSQI Global Score	Sleep Difficulty Classification (SDC)	General Stress Score	General Recovery Score	Sport-Specific Stress	Sport-Specific Recovery
TNK Score (%)	Pearson’s r	—						
	*p*-value	—						
PSQI Global Score	Pearson’s r	−0.08	—					
	*p*-value	0.57	—					
Sleep Difficulty Classification (SDC)	Pearson’s r	−0.21	0.67 ***	—				
	*p*-value	0.13	<0.001	—				
General Stress Score	Pearson’s r	−0.06	0.61 ***	0.33 *	—			
	*p*-value	0.70	<0.001	0.02	—			
General Recovery Score	Pearson’s r	0.01	−0.45	−0.29 *	−0.39 ***	—		
	*p*-value	0.94	<0.001 ***	0.04	<0.001	—		
Sport-Specific Stress	Pearson’s r	−0.04	0.47 ***	0.26	0.61 ***	−0.34 *	—	
	*p*-value	0.78	<0.001	0.06	<0.001	0.02	—	
Sport-Specific Recovery	Pearson’s r	0.01	−0.36 **	−0.21	−0.48 ***	0.64 ***	−0.58 ***	—
	*p*-value	0.92	<0.01	0.15	<0.001	<0.001	<0.001	—

Note. * *p* < 0.05, ** *p* < 0.01, *** *p* < 0.001. TNK = total nutrition knowledge; PSQI = Pittsburgh Sleep Quality Index.

## Data Availability

The datasets presented in this article are not readily available due to the sensitive nature of the data and ethical considerations regarding adolescent participant privacy.
